# CXCL8, CXCL9, CXCL10, and CXCL11 as biomarkers of liver injury caused by chronic hepatitis B

**DOI:** 10.3389/fmicb.2022.1052917

**Published:** 2022-11-24

**Authors:** Xin Yu, Ying Chen, Lele Cui, Kaming Yang, Xumeng Wang, Linyuan Lei, Yanping Zhang, Xinyi Kong, Wanwen Lao, Zhenlin Li, Yang Liu, Yuetong Li, Changlong Bi, Chao Wu, Aixia Zhai

**Affiliations:** ^1^Department of Laboratory Medicine, The Eighth Affiliated Hospital, Sun Yat-sen University, Shenzhen, China; ^2^Department of Immunology, School of Basic Medical Sciences, Capital Medical University, Beijing, China; ^3^Department of Endocrinology, The Eighth Affiliated Hospital, Sun Yat-sen University, Shenzhen, China; ^4^Department of Microbiology, Harbin Medical University, Harbin, China

**Keywords:** hepatitis B virus, CXCL8, CXCL9, CXCL10, CXCL11

## Abstract

**Background:**

Chronic hepatitis B (CHB) remains a significant global health problem, leading to recurrent inflammation and liver-damaging diseases such as fibrosis, cirrhosis, and hepatocellular carcinoma (HCC). Currently, although diagnostic markers for CHB are well established, the indicators for predicting liver injury caused by hepatitis B virus (HBV) infection still need to be further explored. Thus, the identification of credible infectious indicators is urgently needed to facilitate timely clinical intervention and avoid the progression of disease malignancy.

**Methods:**

The Gene Expression Omnibus (GEO) database GSE83148 data set was used to explore the hub genes for HBV infection. The quantitative real-time polymerase chain reaction (qPCR) was used to identify the impact of HBV infection on the expression of hub gene at the cell level. At the same time, serum samples and clinical information were collected from healthy, HBV-free and CHB patients. The enzyme-linked immunosorbent assay (ELISA) was used to verify the results of cell experiments and Pearson correlation analysis was used to clarify hub genes correlation with HBV infection indicators and liver injury-related indicators. Finally, the Gene Expression Profiling Interactive Analysis (GEPIA) database was used to analyze the differences in the expression of hub gene in liver injury diseases.

**Results:**

Chemokine (C-X-C motif) ligand (CXCL)8, CXCL9, CXCL10, and CXCL11 were identified as hub genes in HBV infection. After HBV infection, the expression of the four chemokines was significantly increased and the concentrations secreted into serum were also increased. Moreover, the four chemokines were significantly correlated with HBV infection-related indicators and liver injury-related indicators, which were positively correlated with alanine aminotransferase (ALT), aspartate aminotransferase (AST) and hepatitis B e antigen (HBeAg), and negatively correlated with AST/ALT ratio and hepatitis B core antibody (HBcAb). In addition, the expression of CXCL9, CXCL10, and CXCL11 in HCC tissues was significantly higher than in normal tissues.

**Conclusion:**

Using a combination of bioinformatics, cell experiments, and clinical correlation analysis, this study showed that CXCL8, CXCL9, CXCL10, and CXCL11 can be used as serum biomarkers to forecast liver injury caused by HBV infection.

## Introduction

Hepatitis B virus (HBV) is the most critical factor in acute and chronic hepatitis infection (Yang et al., 2017). Currently, 350 million people worldwide suffer from chronic hepatitis B (CHB), about 120 million of whom are Chinese (Yuen et al., 2018; Gao et al., 2019). CHB, a canonical inflammatory disease, has been identified as the primary cause for the progression of liver-related diseases, which can result in death. After HBV infection, cytokines of the liver microenvironment change, as exemplified by inflammatory cytokine aggregation (Zhong et al., 2021). The expression of chemokines increases, generating a concentration gradient signal that helps in recruiting inflammatory cells such as neutrophils and T lymphocytes to the inflammation parts, triggering an inflammatory reaction. On the one hand, these inflammatory cells assist organisms in eliminating viruses. On the other hand, they constantly destroy liver parenchyma. Consequently, HBV infection lasts over a long period and finally triggers liver damage disease (Kolaczkowska and Kubes, 2013; Mehrfeld et al., 2018; Peng et al., 2020; Zhong et al., 2021).

The World Health Organization (WHO) reported that, in 2019, HBV resulted in an estimated 820,000 deaths, mostly from cirrhosis and hepatocellular carcinoma (HCC; World Health Organization (WHO) Hepatitis B factsheet, 2018). At present, the most effective therapeutic strategy for HCC is hepatectomy, but only a small fraction of patients can be operated on because they were diagnosed with advanced liver cancer (Sugawara and Hibi, 2021). As a result, valid biomarkers are urgently needed in clinical practice to predict CHB-induced liver injury to facilitate timely clinical intervention, preventing the progression of disease malignancy. Therefore, we began with differentially expressed genes in the liver microenvironment to explore biomarkers secreted into the blood that could be used to predict liver damage after HBV infection. Microarray analysis is frequently applied to research the pathogenesis of cancer, but more and more studies concentrate on infectious diseases. Therefore, we downloaded gene chips *via* a Gene Expression Omnibus (GEO) data set to analyze and predict the correlation of four chemokines, expressions with CHB in the liver tissue microenvironment, including chemokine (C-X-C motif) ligand (CXCL)8, CXCL9, CXCL10, and CXCL11. Then, these four chemokines were examined in clinical blood samples to determine whether they could be used as predictors of liver injury caused by HBV infection.

## Materials and methods

### Patients

The Research Ethics Board of the Eighth Affiliated Hospital of Sun Yat-sen University approved this study. Written informed consent was obtained from all patients involved. 93 patients at the Eighth Affiliated Hospital of Sun Yat-sen University from February 2020 to April 2021 provided the serum samples used in this research. In this study, 28 healthy patients, 45 patients with CHB and 20 patients without HBV infection and alanine aminotransferase (ALT) levels comparable to CHB patients were enrolled. The latter served as the disease control group and was named as the HBV-free group. Following the Chinese Society of Liver Diseases and the Chinese Society of Infectious Diseases standards, the criteria for inclusion in the CHB group were positive serum hepatitis B surface antigen (HBsAg) and continuous or repeated elevation of serum alanine aminotransferase for more than 6 months. The HBV DNA, HBsAg, ALT and aspartate aminotransferase (AST) data of the patients were obtained from the laboratory report. HBV DNA was detected by ABIQ5 PCR instrument using quantitative real-time polymerase chain reaction (qPCR). HBsAg was detected by Roche cobas e601 automatic immunoassay system using electrochemiluminescence method with a detection limit of 0.05 IU/ml. ALT and AST were quantified by lactate dehydrogenase method and malate dehydrogenase method, respectively, with the detection limit of 40 U/ml by Beckman AU5800 automatic biochemical analyzer. In addition, the clinical diagnosis was consistent with the “Guidelines for the Prevention and Treatment of Chronic Hepatitis B 2019” excluding the influences of pregnancy, hepatitis C virus (HCV), human immunodeficiency virus (HIV), and other diseases.

### Microarray data

The microarray data for GSE83148 were downloaded from the GEO database^1^ based on the GPL570 [HG-U133_Plus_2] Affymetrix Human Genome U133 Plus 2.0 Array. A total of 128 liver tissues, including 122 HBV infected samples and 6 healthy samples, were used in this study.

### Data processing and differential expression analysis

For the GSE83148 dataset, the original array data was downloaded by the R packages named *GEOquery* and *tinyarray*. Data collating was conducted *via* stringr, an R package. The IDs of genes were converted with the R packages called *AnnoProbe* and *hgu133plus2.db*. Then the differential expression analysis was carried out using an R package named DESeq2 to identify differentially expressed genes (DEGs) between HBV and normal samples and using R package *dplyr* to process the data derived from the above analysis. We used a series of indexes for DEG screening, including a |log2FC| >1 and a *p* value cutoff <0.05. We used the R packages called *ggplot2* and *tinyarray* to create a volcano map and clustering heatmap of DEGs.

We used the Gene Expression Profiling Interactive Analysis (GEPIA) database^2^ to compare the expression of CXCL8, CXCL9, CXCL10, and CXCL11 in normal tissues and HCC tissues by setting the threshold of *p* value to 0.05 and the threshold of |log2FC| to 0.5. The data of GEPIA were obtained from The Cancer Genome Atlas (TCGA^3^) and Genotype-Tissue Expression (GTEx^4^) databases, in which HCC tissue data were obtained from TCGA database, and normal tissue data were obtained from TCGA and GTEx database.

### Go and KEGG pathway analyses

Genome Ontology (GO) analysis is usually used to investigate the annotation of genes and proteins as a bioinformatics tool. Kyoto Encyclopedia of Genes and Genomes (KEGG) is a database used to systematically analyze gene functions, genomic link information, and integrate protein interaction network information. The R packages named *clusterProfiler*, *org.Hs.eg.db*, *GOplot*, *stringr* and *tinyarray* were applied to implement GO enrichment analysis and KEGG annotations. A *p* value cutoff <0.05 was identified as a significant difference.

### PPI network analysis and module analysis

The Search Tool for the Retrieval of Interacting Genes (STRING)^5^ database is a resource to evaluate protein–protein interaction (PPI) network information. Nodes represented genes, and nodes were connected by lines, representing gene relationships in the PPI network. PPI network production was performed with the above top50 DEGs of clustering heatmap. Genes with more than five nodes between them and other genes are defined as the hub genes, which perform essential biological functions in HBV. Cytoscape and cytoHubba were used to analyze the hub genes and map the molecular interaction networks.

### Cell culture

A liver cancer cell line (HepG2) derived from a 15-year-old Caucasian boy is often used to study liver-related diseases. HepG2.215 cells are derived from HepG2 cells and are transfected with four 5′-3′ tandem copies of the HBV genome, usually used as cell models of hepatitis B virus infection (Sells et al., 1987). HepG2 cells and HepG2.215 cells were cultured in DMEM medium, supplemented with 10% FBS and 1% penicillin–streptomycin, and incubated at 37°C in a 5% CO_2_ environment.

### qPCR

According to the manufacturer’s instructions, total RNA was extracted from cells using Trizol reagent (Invitrogen, Carlsbad, CA, United States). One microgram of total RNA from each sample was used to generate cDNAs with the PrimeScript™ RT Master Mix (#RR036A; Takara Bio Inc., Shiga, Japan). The resultant cDNA was amplified by TB Green® Premix Ex Taq™ II (#RR820A; Takara Bio Inc., Shiga, Japan). The primer sequence is shown in Table 1. The program was as follows: holding stage 95°C 30 s; cycling stages 95°C for 5 s, 60°C for 30 s, and 60°C for 30 s, repeated for 50 cycles; and melting stages 95°C for 15 s, 69°C for 60 s, and 95°C for 15 s. The levels of mRNA were normalized relative to GAPDH. The expression of genes was analyzed by using the 2^-△△CT^ method.

**Table 1:** qPCR primer sequences.

Name	Forward primer	Reverse primer
GAPDH	F:GGACCTGACCTGCCGTCTAG	R:GTAGCCCAGGATGCCCTTGA
CXCL8	F:AACTTTCAGAGACAGCAGAGCACAC	R:CACACAGTGAGATGGTTCCTTCCG
CXCL9	F:CAGCACCAACCAAGGGACTATC	R:CACATCTGCTGAATCTGGGTTTAG
CXCL10	F:CTGCCATTCTGATTTGCTGCC	R:AATGCTGATGCAGGTACAGCG
CXCL11	F:GGCAGATATTGAGAAAGCCTCCA	R:GCCTTGCTTGCTTCGATTTGG

### Elisa

The levels of CXCL8, CXCL9, CXCL10, and CXCL11 expression in human serum samples were detected using CXCL8, CXCL9, CXCL10, and CXCL11 enzyme-linked immunosorbent assay (ELISA) kits (CXCL8, YH-10139; CXCL9, YH-10060; CXCL10, 10,080; CXCL11, 10,061, YIHE biological, Shanghai, China), according to the manufacturer’s instructions. Absorbance at 450 nm in each well measured the intensity of the developed color. Standard curves for the assay system were obtained from dilutions of the standards of the ELISA kit. The concentrations of CXCL8, CXCL9, CXCL10, and CXCL11 were then obtained by extrapolation from the standard curve.

### Statistical analysis

Statistical analysis was carried out using SPSS 25 software. Pearson’s correlation coefficient was used to evaluate the correlations among the expressions of four chemokines and the levels of ALT and AST. All statistical data were analyzed and visualized with GraphPad Prism 6.0 software (GraphPad Software, La Jolla, CA). The qPCR validation of all samples was tested by a paired *t* test, and a *p* value < 0.05 was considered statistically significant. All continuous variables were expressed as the means ± SD. Analysis of variance (ANOVA) was used for statistical comparison among groups. All cell experiments were performed in triplicate.

## Results

### Identification of hub genes related to HBV infection

**Figure 1:**
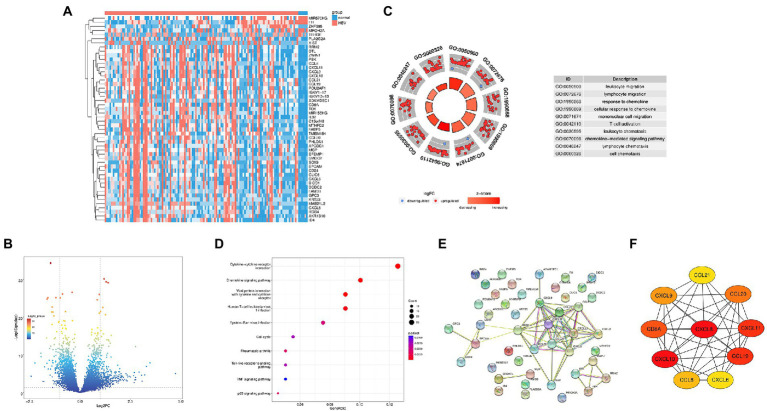
Identification of the Hub genes in HBV infection. **(A)** Heat map of top50 differential genes, with blue representing downregulated genes and pink representing upregulated genes; **(B)** Volcanic plot of differentially expressed genes. **(C)** GO analysis diagrams of differential gene; **(D)** KEGG analysis diagrams of differential genes; **(E)** PPI network for top50 differential genes. **(F)** Interaction network for hub genes.

In the GEO database, we compared the genes expression between the HBV infected samples and normal control samples, 444 DEGs were successfully identified, including 338 upregulated and 106 downregulated genes. We according to the genetic |log2FC| and take the top50 genes for heatmap display (Figure 1A). Figure 1B show the volcano plot of DEGs. To further investigate the biological function of DEGs, GO analysis was performed. The results showed that DEGs were enriched in leukocyte migration, lymphocyte migration, response to chemokine (Figure 1C). Furthermore, KEGG analysis showed that DEGs were concentrated in 10 signaling pathways, including cytokine-cytokine receptor interaction, chemokine signaling pathway, viral protein interaction with cytokines and cytokine receptors (Figure 1D). Based on the STRING database, we performed the PPI network of the top50 DEGs (Figure 1E) and combined it with the KEGG results. CytoHubba was used to map the hub gene network (Figure 1F). Ultimately, we identified four HBV infection-related hub genes: CXCL8, CXCL9, CXCL10, and CXCL11, which were all upregulated.

### HBV infection upregulated the expressions of CXCL8, CXCL9, CXCL10, and CXCL11

**Figure 2:**
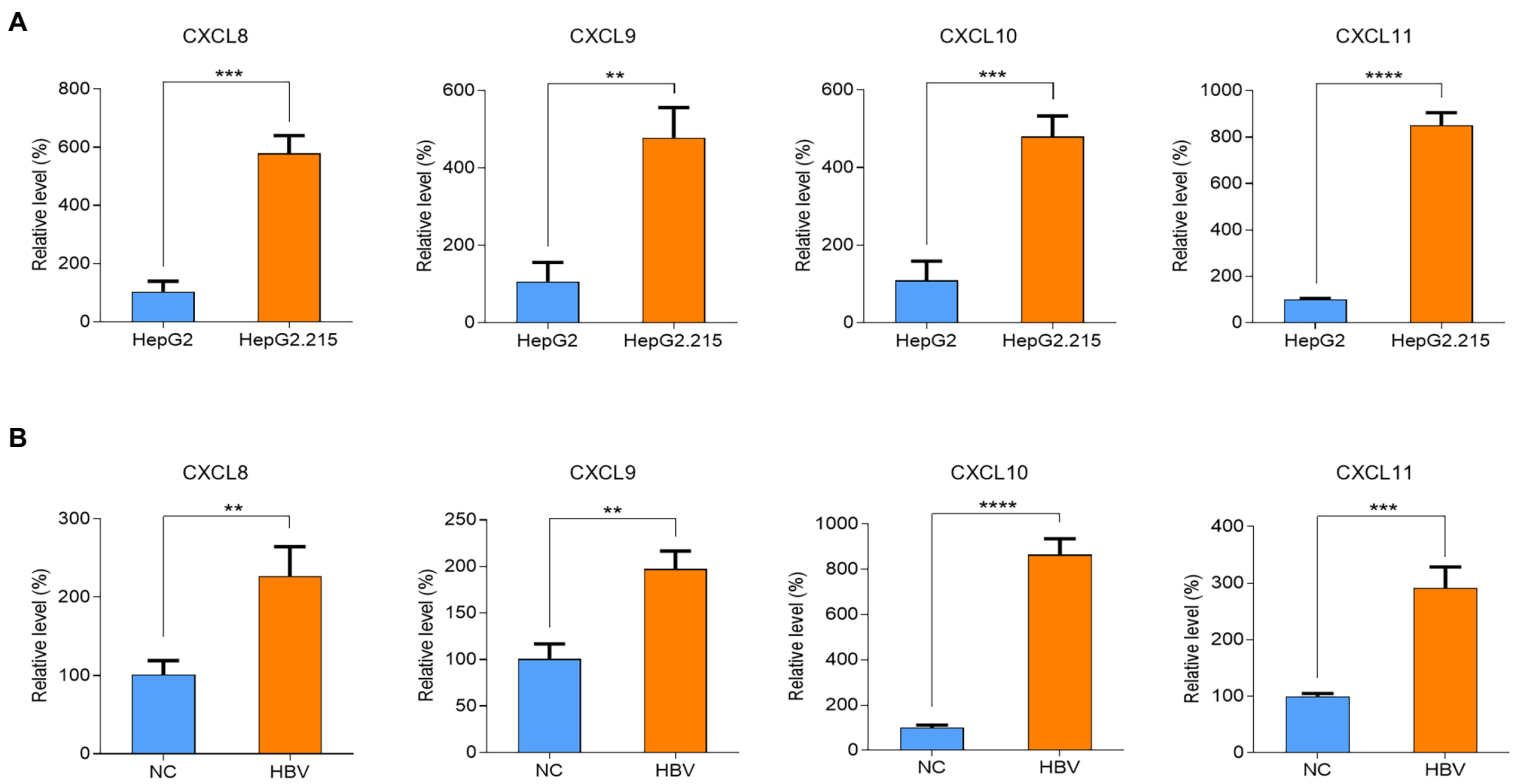
Differences of CXCL8, CXCL9, CXCL10 and CXCL11 mRNA levels after HBV infection. **(A)** Validation of CXCL8, CXCL9, CXCL10 and CXCL11 mRNA levels. **(B)** Validation of CXCL8, CXCL9, CXCL10 and CXCL11 mRNA levels after transfection with HBV plasmid. NC refers to HBV control plasmid, HBV refers for HBV 1.3-mer WT replicon plasmid. ** *p* < 0.01, *** *p* < 0.001, **** *p* < 0.0001.

To validate the relationship between HBV infection and hub genes, we analyzed the mRNA expression of hub genes in HepG2 cells and HepG2.215 cells by qPCR. As shown in Figure 2A, the mRNA levels of CXCL8, CXCL9, CXCL10, and CXCL11 were upregulated in HepG2.215 cells which containing HBV genome compared to HepG2 cells. We also re-verified the above results in the HepG2 cells with transient HBV genome (Figure 2B). On this basis, we conclude that the expression of chemokines CXCL8, CXCL9, CXCL10, and CXCL11 was markedly upregulated after HBV infection.

### Serum levels of CXCL8, CXCL9, CXCL10, and CXCL11 in CHB patients were increased

**Figure 3:**
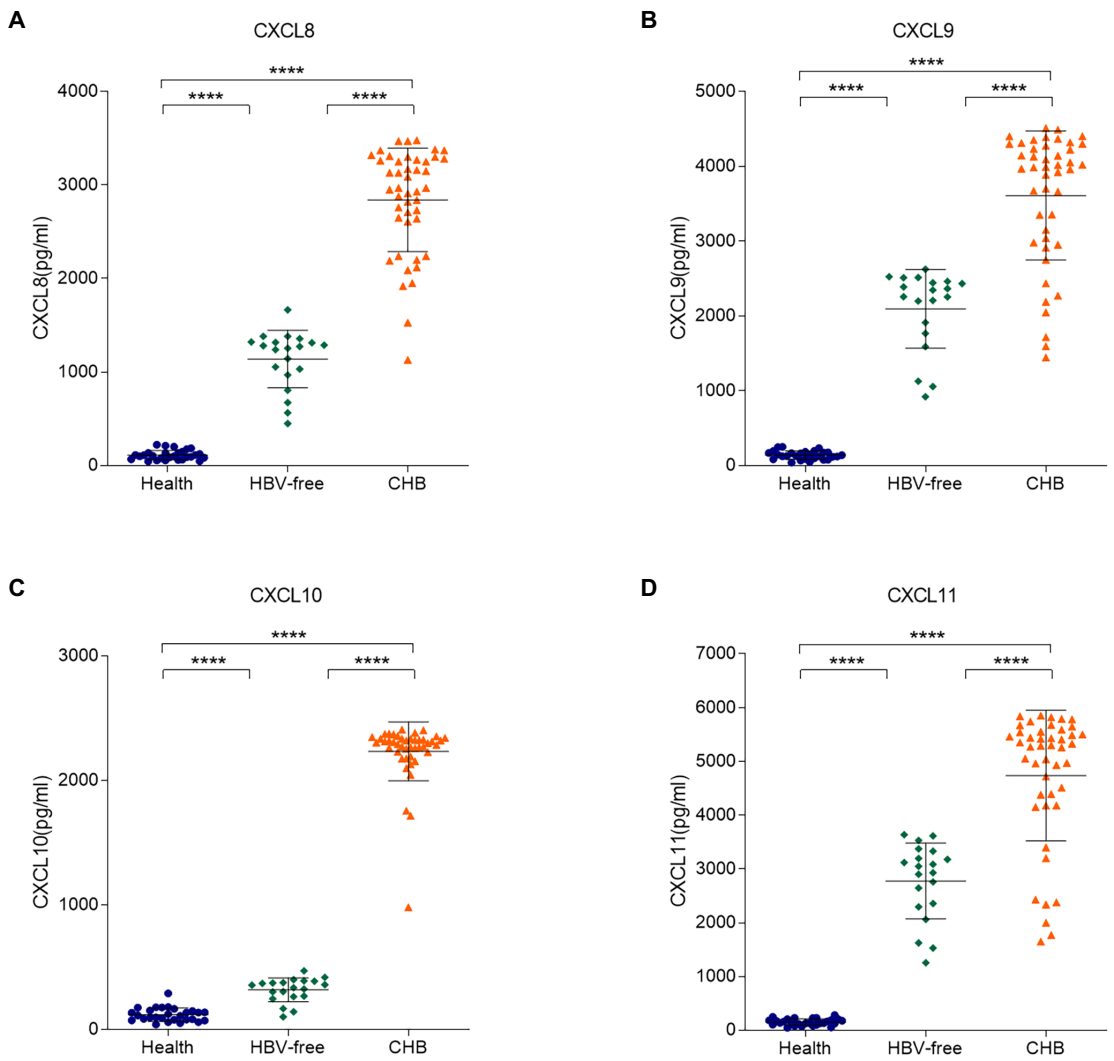
Differences in serum levels of CXCL8, CXCL9, CXCL10, and CXCL11 between the HBV-infected, HBV-free and the Healthy groups. The serum levels of **(A)** CXCL8, **(B)** CXCL9, **(C)** CXCL10, and **(D)** CXCL11 in the Healthy group, the HBV-free group and the HBV-infected group were detected by ELISA. The detection limit of CXCL8, CXCL9, CXCL10, and CXCL11 is 12.5–9,000 pg./ml, 17.8–12,000 pg./ml, 28–3,200 pg./ml and 31–16,000 pg./ml, respectively. **** p < 0.0001.

To further clarify the correlation between these four chemokines in serum samples and HBV infection, ELISA experiments were conducted. We tested the concentrations of the four chemokines in the serum samples from 28 healthy patients, 45 CHB patients and 20 HBV-free patients (Figure 3). Compared with the Healthy group and the HBV-free group, the serum levels of CXCL8, CXCL9, CXCL10, and CXCL11 in patients with the CHB group were significantly higher. Combined with cell experiments, we speculated that the changes of the four chemokines may be detected by blood samples to speculate the occurrence of HBV infection-related liver injury, so as to meet the requirements of simple, efficient and accurate detection in clinical practice.

### CXCL8, CXCL9, CXCL10, and CXCL11 were positively correlated with HBV infection and ALT

**Figure 4:**
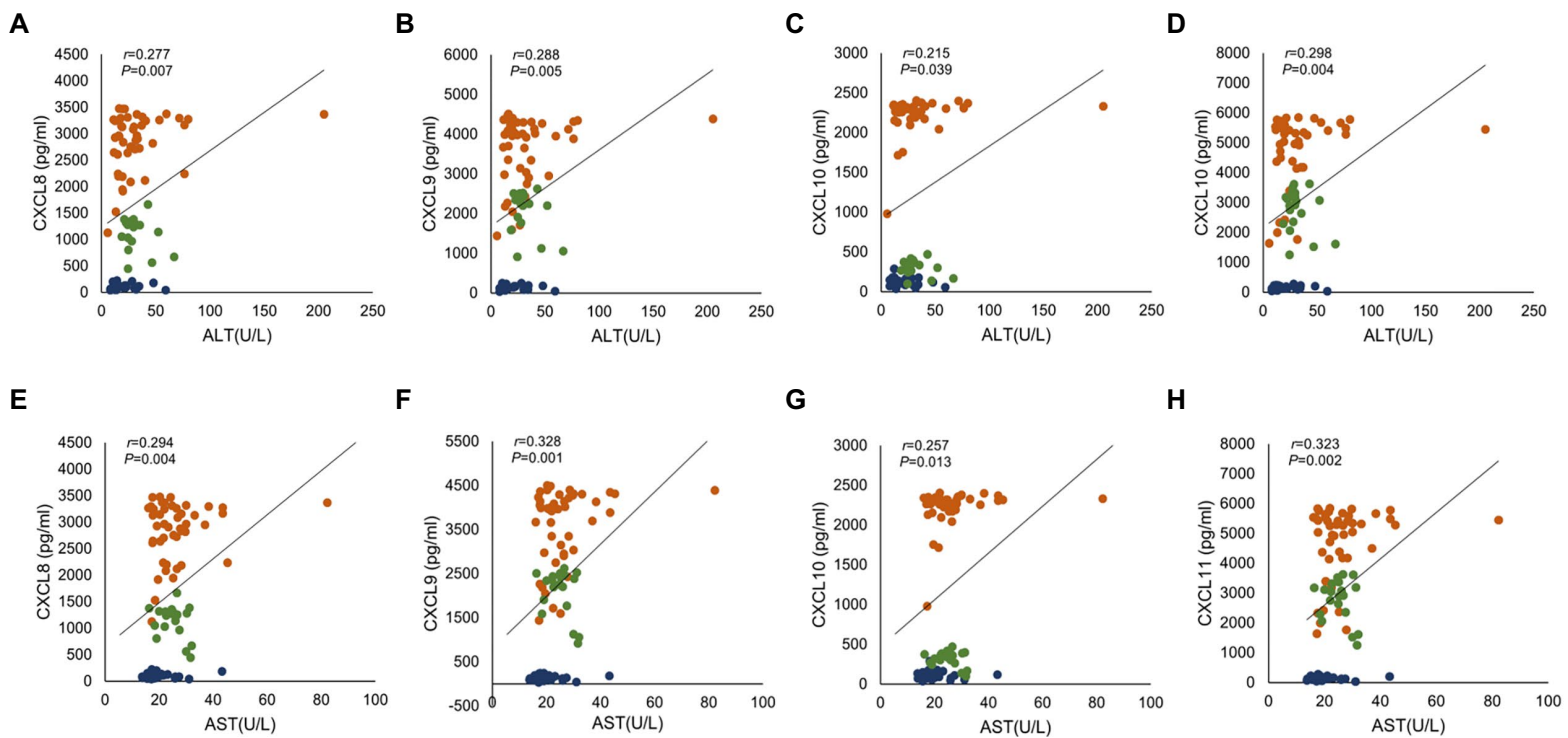
The correlation analysis between serum chemokines and ALT and AST. Association of four chemokines with ALT and AST in 28 healthy subjects, 45 CHB patients and 20 HBV-free patients. Correlation with ALT: **(A)** CXCL8, **(B)** CXCL9, **(C)** CXCL10 and **(D)** CXCL11; Correlation with AST: **(E)** CXCL8, **(F)** CXCL9, **(G)** CXCL10 and **(H)** CXCL11. Orange indicates CHB group, green indicates HBV-free group and blue indicates Healthy group. Comparisons were made using Pearson correlation analysis.

We sorted out the clinical data of Healthy patients, CHB patients and HBV-free patients. ALT is a common marker of hepatocyte injury and inflammation. As show in Table 2, ALT levels between the Healthy group and the CHB group were significantly different, while the difference between the HBV-free group and the CHB group was not statistically significant. We analyzed the clinical association between these four factors and ALT and AST using the Pearson correlation analysis. As shown in Figure 4; Table 3, CXCL8, CXCL9, CXCL10, and CXCL11 showed positive correlation with ALT, AST, hepatitis B e antigen (HBeAg), and negatively correlated with AST/ALT ratio, hepatitis B core antibody (HBcAb). In addition, the results indicated that both CXCL8 and CXCL10 were positively correlated with total bilirubin (TBIL) and indirect Bilirubin (IBIL; Table 3). These results suggested that the changes of CXCL8, CXCL9, CXCL10, and CXCL11 are significantly positive association with HBV infection and indicators related to traditional liver injury related indicators, indicating that these four factors can be used as potential specific predictors of liver injury caused by HBV infection.

**Table 2:** Comparison of differences between the Healthy group, the HBV-free group and the CHB group.

Indexes	Healthy group(*n* = 28)	HBV-free group(*n* = 20)	CHB group(*n* = 45)	Healthy vs. CHB*t* value	Healthy vs. CHB*p* value	HBV-free vs. CHB*t* value	HBV-free vs. CHB*p* value
Age	39.36 ± 1.4500	43.25 ± 2.8040	35.73 ± 1.4930	1.638	0.1059	2.581	0.0122
TBIL	13.23 ± 0.6905	11.32 ± 0.9489	15.78 ± 1.1730	1.612	0.1113	2.381	0.0203
DBIL	2.38 ± 0.1391	2.31 ± 0.2913	2.73 ± 0.1860	1.349	0.1815	1.245	0.2179
IBIL	10.85 ± 0.5816	8.80 ± 0.7396	13.05 ± 1.0030	1.629	0.1077	2.676	0.0095
TP	71.40 ± 0.7522	69.99 ± 1.7410	71.31 ± 0.6065	0.0951	0.9245	0.8957	0.3738
ALB	42.31 ± 0.3893	42.12 ± 0.9505	42.52 ± 0.3661	0.3694	0.7129	0.4829	0.6308
γ-GT	37.07 ± 6.5700	32.28 ± 3.7140	29.70 ± 3.4390	1.089	0.2799	0.4497	0.6545
ALT	20.01 ± 2.3380	31.51 ± 2.6540	33.57 ± 4.7790	2.137	0.036	0.2779	0.782
AST	20.36 ± 1.1490	25.08 ± 1.0280	26.33 ± 1.6710	2.585	0.0118	0.4801	0.6328
AST/ALT	1.21 ± 0.0766	0.85 ± 0.0459	1.05 ± 0.0762	1.421	0.1598	1.656	0.1027
GLB	29.09 ± 0.6338	27.80 ± 0.9000	28.79 ± 0.4340	0.4011	0.6896	1.119	0.2673
A/G	1.48 ± 0.0355	1.53 ± 0.0491	1.49 ± 0.0247	0.384	0.7021	0.8495	0.3988
HBeAg	0.12 ± 0.0017	0.10 ± 0.0021	179.90 ± 63.8200	2.216	0.0299	1.87	0.0661
HBeAb	1.21 ± 0.0966	1.25 ± 0.1408	0.89 ± 0.2505	0.9641	0.3383	0.9153	0.3635
HBcAb	1.51 ± 0.2016	1.29 ± 0.2546	0.05 ± 0.0364	8.849	<0.0001	7.042	<0.0001
CXCL8	112.80 ± 9.5560	227.8 ± 13.7800	2,842 ± 82.5600	25.94	<0.0001	20.96	<0.0001
CXCL9	140.40 ± 10.6700	2096 ± 117.7000	3,609 ± 128.6000	21.2	<0.0001	7.252	<0.0001
CXCL10	118.70 ± 10.1700	317.9 ± 21.4100	2,234 ± 35.2300	46.5	<0.0001	34.88	<0.0001
CXCL11	153.10 ± 11.5500	2,774 ± 157.8000	4,735 ± 181.4000	19.86	<0.0001	6.709	<0.0001

Comparisons were made using *t*-test analysis.

TBIL, total bilirubin; DBIL, direct bilirubin; IBIL, indirect bilirubin; TP, total protein; ALB, albumin, γ-GT γ-glutamyl transpeptidase; ALT, alanine aminotransferase; AST, aspartate transaminase; AST/ALT, ratio of aspartate aminotransferase to alanine aminotransferase; GLB, globulin, A/G ratio of albumin to globulin; HBeAg, hepatitis B e antigen; HBeAb, hepatitis B e antibody; HBcAb, hepatitis B core antibody.

**Table 3:** Correlation analysis of serum chemokines expression levels with biochemical indexes and HBV infection related indexes.

	CXCL8		CXCL9		CXCL10		CXCL11	
	*r*	*p*	*r*	*p*	*r*	*p*	*r*	*p*
AST/ALT	**−0.211**	0.042	**−0.219**	0.035	−0.101	0.333	**−0.246**	0.018
TBIL	**0.238**	0.022	0.158	0.13	**0.274**	0.008	0.189	0.07
DBIL	0.19	0.068	0.139	0.185	0.194	0.062	0.168	0.107
IBIL	**0.242**	0.019	0.156	0.134	**0.287**	0.005	0.185	0.076
HBeAg	**0.288**	0.005	**0.249**	0.016	**0.306**	0.003	**0.205**	0.048
HBcAb	**−0.575**	<0.0001	**−0.521**	<0.0001	**−0.638**	<0.0001	**−0.512**	<0.0001

Association of four chemokines with biochemical indexes and HBV infection related indexes in 28 healthy subjects, 45 CHB patients and 20 HBV-free patients. Comparisons were made using Pearson correlation analysis.

ALT, alanine aminotransferase; AST, aspartate transaminase, AST/ALT, ratio of aspartate aminotransferase to alanine aminotransferase; TBIL, total bilirubin; DBIL, direct bilirubin; IBIL indirect bilirubin; HBeAg, hepatitis B e antigen; HBcAb, hepatitis B core antibody.

Bold values indicate that the group of comparisons is statistically significant.

### The expression of CXCL9, CXCL10, and CXCL11 was elevated in HCC

**Figure 5:**
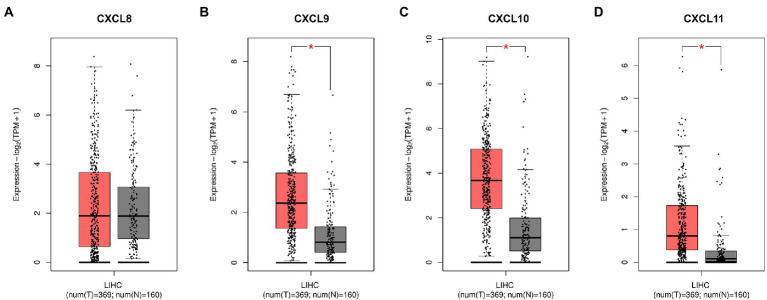
The differences of serum chemokines between normal tissues and HCC tissues. Differences in **(A)** CXCL8, **(B)** CXCL9, **(C)** CXCL10, and **(D)** CXCL11 levels between normal tissues and HCC tissues, based on GEPIA database. HCC tissue is shown in red and normal tissue is shown in gray. Expression-log_2_ (TPM + 1) indicates the expression level of corresponding genes in HCC tissues and normal tissues. Asterisk (*) in red indicates *p* < 0.05.

HBV infection can cause inflammation, and if the patient is not treated effectively, repeated inflammation can lead to further liver damage, progressing to HCC. Therefore, based on GEPIA database, we analyzed the expressions of CXCL8, CXCL9, CXCL10, and CXCL11 in normal tissues and HCC tissues. As shown in Figure 5, CXCL9, CXCL10 and CXCL11 were markedly upregulated in HCC tissues compared with normal tissues, and the differences were statistically significant. In conclusion, CXCL9, CXCL10, and CXCL11 may play a warning role in the early stage of liver injury, so as to prevent the further aggravation of injury and the development of HCC.

## Discussion

In recent years, distorted cytokine expression has been found in acute and chronic hepatitis B patients (Ribeiro et al., 2022). Our study found that CXCL8 was disregulated in HBV infection. In addition, the chemokines CXCL9, CXCL10, and CXCL11 were also predicted to play an important role in HBV infection. Chemokines are necessary for leukocyte migration during inflammation, and are closely related to the localization of leukocytes in lymphatic follicles and specific organs and tissues (Vanheule et al., 2015). Chemokines are mainly induced by inflammatory cytokines, growth factors, and pathogenic microorganisms (Reid-Yu et al., 2015), which can be divided into four subtypes: C, CC, CXC, and CX3C, according to the position of the NH2 terminal cysteine (Hughes and Nibbs, 2018). CXCL8, CXCL9, CXCL10, and CXCL11 are members of the CXC family. They are the inflammatory chemokines that participate in cell migration and the inflammatory response.

CXCL8, also known as interleukin 8 (IL-8) and neutrophil activator (NAF), is a multifunctional proinflammatory chemokine that activates neutrophils and sends recruitment signals through concentration gradients to recruit activated neutrophils to inflammatory sites (Xu et al., 2016; Asokan and Bandapalli, 2021). Many studies have shown that high levels of CXCL8 can be detected in the serum of HBV-infected patients (Pollicino et al., 2013). HBV can directly target the CXCL8 promoter and regulate the expression of CXCL8 through epigenetic modifications, but the protein of hepatitis B virus X (HBx) encoded by HBV can promote the increase of CXCL8 expression through the MEK–ERK signal pathway (Pollicino et al., 2013; Zhang et al., 2021). We used an HBV stable and transient cell model to detect the relationship between HBV and CXCL8 expression. The results showed that CXCL8 expression was significantly upregulated in the presence of the HBV genome. We further analyzed CXCL8 expression in serum samples from CHB patients, and found that the levels of CXCL8 released into the serum was significantly higher in CHB patients than in Healthy group. To investigate the possibility of CXCL8 as a predictor of liver-damaging diseases caused by HBV infection, we conducted a correlation analysis between CXCL8 and liver injury related indexes (ALT, AST), which showed a positive correlation. All these results confirmed that CXCL8 could be used to predict liver-damaging diseases caused by HBV infection.

Our results showed that there was no difference in CXCL8 expression between normal tissues and HCC tissues, but we confirmed that HBV infection may increase the expression of CXCL8 in both cell experiments and clinical blood samples. In addition, studies have shown that CXCL8 is related to the occurrence and development of HCC (Xu et al., 2021). HBV-induced CXCL8/CXCR1/TGF-β signaling cascade can mediate HCC vascular invasion and local microenvironment immune escape to induce intrahepatic metastasis of HCC (Zhang et al., 2021). CXCL8 can also promote up-regulation of integrin β3 and enhance the invasion ability of HCC cells through the PI3K/AKt pathway (Sun et al., 2019). In conclusion, CXCL8 may plays an important role in the occurrence and development of liver injury diseases caused by HBV infection.

It has been reported that CXCL9, CXCL10, and CXCL11 all play an important role in HBV infection and exert signaling functions through C-X-C motif chemokine receptor 3 (CXCR3) receptors (Yoshio et al., 2018). Studies have shown that these three chemokines correlate significantly with serum alanine aminotransferase (Lian et al., 2014). Their main function is chemotactic, recruiting immune cells to sites of inflammation for repair and enhancing the T-cell response to viral infection (Macura et al., 2016; Vanheule et al., 2017; Marcovecchio et al., 2021). Several studies have shown that CXCL9, CXCL10, and CXCL11 are associated with HBV infection. For example, HBx binds to its promoter by activating NF-κB, which induces CXCL9 transcription and promotes leukocyte migration to the HBV-infected liver (Xia et al., 2009). CXCL10 was found to be the strongest signal marker that can be used to characterize the stage of chronic HBV infection (Wiegand et al., 2019). The increase in CXCR3-related chemokines (including CXCL9, CXCL10, and CXCL11) may be associated with the migration of tumor necrosis factor-associated apoptosis-inducing ligand CD56 natural killer (NK) cells to the liver (Jiang et al., 2021).

In addition, these three chemokines play a key role in liver-damaging diseases such as hepatocellular carcinoma. Tumor-associated dendritic cells can produce high levels of CXCL9, which promotes tumor progression by increasing programmed death-ligand 1 (PD-L1) expression through the activation of CXCR3-related signals (Marcovecchio et al., 2021). Transwell assays showed that silencing CXCL10 significantly inhibited the migration of MHCC97H cells, suggesting that CXCL10 promotes the invasion and metastasis of HCC cells (Srivastava et al., 2017). Moreover, CXCL11 is highly associated with tumor formation, progression, metastasis, and angiogenesis, as well as with the adverse effects of chemotherapy (Bandapalli et al., 2012). Cancer-associated fibroblasts secrete significantly higher levels of CXCL11 than normal fibroblasts (NFs), and CXCL11 expression is significantly elevated, indicating that CXCL11 can promote HCC cell migration (Liu et al., 2021). It has been shown that CXCL9, CXCL10, and CXCL11 expression increases after HBV infection, which positively correlates with ALT levels (Jiang et al., 2021). In addition, CXCL9, CXCL10, and CXCL11 are related to the migration of TRAIL CD56^+bright^ NK cells that promote liver injury to the liver in chronic hepatitis B-associated cirrhosis (Lian et al., 2014; Okuhara et al., 2014; Yoshio et al., 2018; Jiang et al., 2021).

ALT and AST are probably the most commonly used biomarkers in clinical diagnosis and research involving liver injury, they catalyze the transfer of an amino group from an amino acid to α-ketoglutarate and the reaction products are L-glutamate and either pyruvate or oxaloacetate, respectively (McGill, 2016). AST is mainly present in myocardium, followed by liver. The exclusive production site of ALT is liver, where the content of ALT was the highest (Kolahdoozan et al., 2020). It is generally thought that aminotransferase elevations are due to cell damage with plasma membrane disruption (Kolahdoozan et al., 2020). When liver tissue damage or destruction increases or cell membrane permeability changes, ALT leaks into the circulation, leading to the increase of the expression level of ALT factor in serum (Nguyen et al., 2014). Therefore, ALT and AST, initially considered markers of liver damage, are increasingly being considered indicators of “liver metabolic function.” (Sookoian and Pirola, 2012; Sookoian and Pirola, 2015). Kechagias et al. (2008) showed that fast-food-based hyper-alimentation in combination with a sedentary lifestyle resulted in elevated serum ALT levels. Moreover, the authors showed that this elevated transaminase is not associated with the development of hepatic steatosis. This finding reinforces the hypothesis that increased ALT activity is an adaptive response to the metabolic demands of the liver (Sookoian and Pirola, 2012, 2015). Therefore, there is an urgent need to explore new and reliable biomarkers of liver injury. In order to reflect the advantages of CXCL8, CXCL9, CXCL10, and CXCL11 in HBV infection-related liver injury compared with ALT, we set the HBV-free group as the disease control group. The subjects of HBV-free group were chronic hepatitis patients without HBV infection and whose ALT levels were comparable to those of patients in the CHB group, including drug-induced liver injury, autoimmune liver disease, alcoholic liver disease and so on. The results showed that CXCL8, CXCL9, CXCL10, and CXCL11 was slightly increased in the HBV-free group compared with the Healthy controls. It was considered that the possible cause was the presence of a certain degree of liver inflammation in patients in the HBV-free group. Therefore, some inflammatory factors in serum are elevated, which is consistent with the reports in the report (Seki and Schwabe, 2015; Czepielewski et al., 2017). However, in this study, serum levels of CXCL8-11 were significantly higher in CHB patients compared to Healthy controls and HBV-free groups. Therefore, CXCL8, CXCL9, CXCL10, and CXCL11 has obvious advantages in HBV-related liver injury compared with traditional ALT markers, suggesting the importance of these four chemokines as a liver injury associated with HBV infection.

In addition, Du et al. found that the bilirubin level in HBsAg-positive group was higher than that in HBsAg-negative group (Du et al., 2016). Consistent with the findings in this report, we found significant differences in TBIL and IBIL between HBV-free and CHB groups. TBIL and IBIL expression was found to be elevated in liver injury (Yang et al., 2016). Our results showed that TBIL and IBIL expression was significantly higher in the CHB group compared to the HBV-free group. In HBV infection, ALT conveys limited information, which can only indicate the liver injury caused by viral factors. However, the result of HBV infection is not only dependent on viral factors, but also related to host factors, such as the immune response of the body (Ribeiro et al., 2022). CXCL8, CXCL9, CXCL10, and CXCL11 can recruit immune cells to the site of inflammation to play a role in clearing pathogens. When inflammation persists, these inflammatory cells will aggravate liver parenchymal cell damage. Therefore, the changes in the expression of these four chemokines can not only reveal the liver injury caused by the virus, but also reflect the strength of the immune function of patients, which has a good indication for the direction of the prognosis of inflammation caused by HBV infection and inflammation-related complications.

Importantly, recurrent inflammation caused by CHB can promote hepatocarcinogenesis. The association between tumors and chemokines has been a hot topic in recent studies on the mechanism of inflammatory cancer transformation (Greten and Grivennikov, 2019; Hou et al., 2021). Based on the results, it is hypothesized that the expression of chemokines CXCL8, CXCL9, CXCL10, and CXCL11 increases after HBV infection, and these four chemokines signal the inflammation as the center and build a concentration gradient to promote the migration of neutrophils and immune cells to the inflammation site to clear the damaged liver parenchyma cells. However, when there is long-lasting inflammation, neutrophils and immune cells are continuously recruited, causing damage to the liver parenchyma, eventually leading to serious irreversible hepatocellular carcinogenesis. Previous studies only focused on a single factor. Our results are the first to link these four chemokines to HBV infection, revealing that all four chemokines are elevated in HBV infection and can be used as predictors of liver-damaging diseases caused by HBV infection. As a key member of the innate immune system, NK cells are the main lymphocytes in liver and play an important role in the immunity of liver diseases (Sun et al., 2015). Chemokines CXCL8, CXCL9, CXCL10, and CXCL11 can promote the directed migration of NK cells during inflammation (Robertson, 2002). In addition, previous studies have found that NK cells can produce CXCL8 factor after activation to promote the chemotaxis of T cells (Robertson, 2002). CXCL10 can enhance the cytolytic activity of NK cells by promoting the release of cytotoxic particles (Taub et al., 1995). Therefore, we hypothesized that the elevated expression of these four chemokines in liver injury caused by HBV infection may be related to the recruited NK cells. However, its specific molecular mechanism and the synergistic effect of these four chemokines in HBV infection remains to be explored. Therefore, we will continue to investigate the synergistic effect of the four chemokines in HBV infection and the mechanism of exacerbating the inflammatory response.

In conclusion, chemokines participate in various cellular processes and the regulation of viral infection, leading to inflammatory injury. Therefore, CXCL8, CXCL9, CXCL10, and CXCL11 are expected to become reliable predictors of liver-damaging diseases caused by HBV infection to facilitate timely intervention measures in clinical treatment and avoid further aggravation of liver injury.

## Data availability statement

The original contributions presented in the study are included in the article/supplementary material, further inquiries can be directed to the corresponding authors.

## Ethics statement

The studies involving human participants were reviewed and approved by the Research Ethics Board of the Eighth Affiliated Hospital of Sun Yat-sen University. The patients/participants provided their written informed consent to participate in this study.

## Author contributions

XY, YC, and LC: data analysis and manuscript writing. KY: data analysis. XW, LL, and YZ: data collection and management. XK, WL, ZL, YaL, and YuL: data collection. CB: data collection and grant support. CW: data management. AZ: manuscript writing and editing, grant support. All authors contributed to the article and approved the submitted version.

## Funding

This work was supported by the National Natural Science Foundation of China (82072267, 82272271, 82172215, and 81871562), Science and Technology Project of Guangdong Province (2021A1515011396 and 2021A1515012109) and Shenzhen Futian District Public Health Research Project (FTWS2020017 and FTWS2020008).

## Conflict of interest

The authors declare that the research was conducted in the absence of any commercial or financial relationships that could be construed as a potential conflict of interest.

## Publisher’s note

All claims expressed in this article are solely those of the authors and do not necessarily represent those of their affiliated organizations, or those of the publisher, the editors and the reviewers. Any product that may be evaluated in this article, or claim that may be made by its manufacturer, is not guaranteed or endorsed by the publisher.

## Footnotes

^1^ http://www.ncbi.nlm.nih.gov/geo/

^2^ http://gepia2.cancer-pku.cn/#analysis

^3^ https://tcga-data.nci.nih.gov/tcga/

^4^ https://www.gtexportal.org/

^5^ https://cn.string-db.org
